# Better methods, better data: landscaping the priorities for improving methodologies in vector control

**DOI:** 10.12688/gatesopenres.15399.2

**Published:** 2025-07-28

**Authors:** Katherine Gleave, Giorgio Praulins, Rosemary Susan Lees

**Affiliations:** 1 Innovation to Impact (I2I), Liverpool School of Tropical Medicine, Liverpool, Merseyside, UK; 2 Vector Biology Department, Liverpool School of Tropical Medicine, Liverpool, UK

**Keywords:** Methods validation, Vector control, Insecticide resistance, Product evaluation

## Abstract

This article addresses the evolving challenges in evaluating insecticide-based tools for vector control. In response to the emergence of insecticide resistance in major malaria vectors, novel chemistries and products are coming to market, and there is a need to review the available testing methodologies. Commonly used methods for evaluating insecticides, such as the World Health Organization (WHO) cone bioassay, are inadequate for the diverse range of tools now available. Innovation to Impact (I2I) has studied the variability in laboratory methods, with the aim of identifying key factors that contribute to variation and providing recommendations to tighten up protocols. The I2I Methods Landscape is a living document which presents a review of existing methods for evaluating vector control tools, with the scope currently extending to insecticide-treated nets (ITNs) and indoor residual sprays (IRS). The review reveals a lack of validation for many commonly used vector control methods, highlighting the need for improved protocols to enhance reliability and robustness of the data that is generated to make decisions in product development, evaluation, and implementation. A critical aspect highlighted by this work is the need for tailored methods to measure endpoints relevant to the diverse modes of action of novel insecticides. I2I envisage that the Methods Landscape will serve as a decision-making tool for researchers and product manufacturers in selecting appropriate methods, and a means to prioritise research and development. We call for collective efforts in the pro-active development, validation, and consistent implementation of suitable methods in vector control to produce the data needed to make robust decisions.

## Disclaimer

The views expressed in this article are those of the author(s). Publication in Gates Open Research does not imply endorsement by the Gates Foundation.

## Purpose

This letter highlights the critical need for updated methodologies for the assessment of vector control tools due to the emergence of insecticide resistance and the development of novel chemistries. Traditional evaluation methods are deemed inadequate for the diverse range of tools now available, necessitating the validation and adoption of innovative approaches. Key initiatives such as the production of a Methods Validation Framework and the Methods Landscaping project by Innovation to Impact (I2I) aim to provide guidance on method reliability, reproducibility, and comparability across different settings. I2I have highlighted priority areas which include validation of established methods, adapting established methods for novel chemistries and developing new methods as needed. Collaboration within the research community is crucial for addressing these challenges and ensuring reliable data is collected for informed decision making in vector control.

## Introduction

Insecticide-treated nets (ITNs) and indoor residual spraying (IRS) are the cornerstone of vector-borne disease control. However, the emergence and spread of insecticide resistance within the major malaria vectors has prompted the development of novel chemistries and products. Methodologies and guidelines that were previously considered robust for evaluating and monitoring pyrethroid-only ITNs against pyrethroid-susceptible mosquito populations have proven inadequate for the diverse range of tools now available. In this rapidly evolving field, it is imperative that we consider the appropriateness of chosen testing methods and that we are able to adopt innovative, robust assessment frameworks.

## Current methods and challenges

The World Health Organisation (WHO) cone and tube bioassays ( World Health Organization, 2013) are widely recognised methods for evaluating the efficacy of insecticides and insecticide-based tools against mosquitoes and for monitoring for insecticide resistance, respectively. These assays involve exposing mosquitoes to treated surfaces and monitoring their knockdown and mortality over a specified time period. The outcomes of these assays provide information about the insecticides’ bioefficacy, which in turn informs decision-making about intervention use for vector control. Over recent years, efforts are starting to be made to validate these and other methods, to ensure their reliability, reproducibility and comparability across different laboratories, geographical settings, and testing environments. The WHO provides guidelines for conducting these assays, including recommendations for parameters such as mosquito species, mosquito age, and insecticide concentration. The efficacy and results of a bioassay depend on the interplay of multiple factors: the test system (mosquito species, resistance status, anthropophagy, physiological status), the bioassay (surface area, duration of exposure, time of day, insect density, insect activity), the environment (temperature and humidity, air flow, ambient light), and the product (active ingredient, formulation, preparation, storage conditions). To fully understand methods and reliably interpret the data they generate, each of these parameters must be investigated. A Methods Validation Framework ( Matope *et al.*, 2023) was designed to optimise and characterise methods and includes an assessment of the effect of altering testing parameters. The application of this process increases confidence in existing and standard testing methods. It is also critical for adapting existing methods to new types of tools and for evaluating new methods developed for emerging needs and novel approaches.

In selecting an appropriate bioassay to generate the data we need to answer a given question, the first thought we need to explore is what the purpose of this method is and whether it is the correct method to use with the product I am testing. We can then consider the factors that will affect reproducibility and robustness, how the method can be used within studies, and whether the method process is sufficiently explained. We should explore the potential sources of variability and bias that need to be monitored and documented. A good example of an investigation into the effects of altering a method parameter comes from a study by Owusu and Muller ( Owusu & Muller, 2016), who considered the angle at which the WHO cone is placed during testing. In this study, the angle at which the cone is mounted significantly affected the amount of time mosquitoes spent resting on treated nets, and hence subsequent mortality. Innovation to Impact (I2I) are currently undertaking studies to investigate the level of variability (referred to as ‘noise’) surrounding the most commonly used laboratory methodologies. This involves looking at repeating bioassays on a large scale to characterise variability under standard conditions, with variables such a relative humidity and mosquito number permitted to fluctuate as they would under routine testing (within the range of acceptable values outlined by WHO guidelines). The purpose of quantifying this day-to-day variation in outcomes is twofold, first to identify which factors are the largest sources of variation, after which we can make recommendations to tighten up the protocol. Secondly, it is important to understand the inherent variability in bioassay data so that testing can be designed to be sufficiently powered to detect real differences between treatments above the inherent noise from the bioassay. Methods can be optimised to minimise noise during internal validation of a method, but it is also important that multi-centre validation is done to confirm inter-laboratory variability. Once validated, a method must be used consistently within studies and between facilities, with any deviations from standardised protocols being reported.

## A new methods landscape

I2I have been surveying the current methods available for assessing vector control tools, during development, evaluation, post market monitoring and insecticide resistance monitoring, and in the vector control field more generally. A Methods Landscape has been produced which characterises and assesses the level of validation of available methods, a living document freely available to the community ( Innovation to Impact, 2024a). The ultimate aim of this Landscape is to serve as a decision-making tool for researchers and product manufacturers to aid in selecting the best methods to generate the right data to answer a given question. By undertaking this exercise, we have been able to identify priority activities and gaps in the evidence needed to ensure better methods are made available for vector control product evaluation. We therefore envisage that this Methods Landscape will also be used as a tool to prioritise investment in appropriate method development and validation by stakeholders including product developers, donors, and researchers.

Undertaking a comprehensive review of existing literature, using available data, and gaining insights from subject matter experts, we have collated an evidence base to support published methods. Through a series of detailed reports, we have explained how best to interpret and use the data generated from different methodologies. A key source of guidance in generating the Landscape was a consultation which aimed to collect information from product developers and manufacturers on their experiences with vector control product evaluation and the methods used to generate data for this purpose. 29 companies were invited to participate, based on their membership of the I2I industry group ( Innovation to Impact, 2024b) at the time of inviting. 13 companies contributed (12 through interview, 1 through written response) responses to a list of pre-provided questions around suitability of available methods, data requirements, endpoints of interest and challenges in data generation. The report is available on the I2I website ( Innovation to Impact, 2024a).

A wide range of factors have been considered in order to characterise methods, including the context for the method (the specific test item for which the method is tailored, suitable chemistries for use with the method, relevant stage of the product’s life cycle, outstanding gaps in knowledge and priorities), the endpoints and their significance (mortality or sterilisation, speed of action, whether entomological effect is relevant to personal or community protection), the relevant testing parameters to consider (the characteristics of the mosquito population used, number of mosquitoes per replicate, exposure and holding times, appropriate controls) and other considerations relevant to method choice (accessibility, cost). For each method, we give guidance on the level of validation, for example is it a standard method for which there is a large evidence base but no formal validation efforts; a newly developed method for a given mode of action chemistry which requires further evaluation; or an established method which has undergone thorough optimisation and formal validation?

Phase one of the Methods Landscape has been launched on the I2I website, which explores 10 methods that I2I have reviewed for evaluating ITNs and IRS. The majority of the methods examined during the landscaping project to date present vague parameters, and protocols need to be improved to increase reliability and robustness. Important aspects of many bioassays such as mosquito age, mosquito number per replicate and the number of biological replicates per product tested require validation, though the updated WHO Guideline for Prequalification Assessment of ITNs and implementation guidance documents ( World Health Organisation, 2024) provide improved clarity.

There has generally been little validation of vector control methods, with that there is being either piecemeal research activities to explore specific parameters or adaptations for a given setting or collected from years of historical data across many different sites using different mosquito species. For many methods in common use there is data available which has been generated across a multi-centre study which can be used to ‘retroactively validate’ a method. Perhaps unsurprisingly, the method with the most data available is the WHO cone test, for which the vast number of published studies using this bioassay offers an opportunity to examine the variability of results and the influence of key endpoints on mortality data, an effort which is underway within I2I. Information may also be gathered to help inform the adaptation of the method for novel chemistries. Another approach to improving and standardising procedures for existing methods is the generation of consensus standardised operating procedures (SOPs), a process I2I has successfully applied to methods for strain characterisation and ITN durability monitoring ( Lees *et al.*, 2022; Lissenden *et al.*, 2021). The most thoroughly validated method reviewed to date is also the newest, the WHO bottle bioassay for susceptibility monitoring, which was optimised through a multi-centre process and with the use of a modelling framework used to generate and confirm discriminating concentrations ( Corbel *et al.*, 2023; Kont *et al.*, 2023). For novel methods, the availability of a Methods Validation Framework ( Matope *et al.*, 2023) provides a formal process that can be followed to generate a specific Methods Claim which characterises the scope and accuracy of outputs of the method and maximise confidence in method use.

## Future direction and focus areas

I2I plan for the Methods Landscape to be a living document, updated as we continue our review and validation of methods, updated to reflect current best practices and evolving evidence and use practices, and expanding the scope from ITNs and IRS to methods related to other product types such as spatial repellents and ATSBs. The Landscape will also incorporate areas beyond purely product evaluation such as screening for novel active ingredients and durability monitoring

Resistance monitoring methods are also an important area for consideration. Current methods used to monitor mosquito populations for insecticide susceptibility were designed for a world where resistance was only beginning to emerge, to detect the first signs of the appearance of resistance mechanisms primarily to pyrethroids. New methods are needed to better characterise resistance and relate that information to operational decisions about product deployment in a new world of widespread resistance. This new method development needs to consider novel modes of action and routes of exposure including ingestion and exposure to volatiles to be relevant for new types of vector control tools. At the same time there are improvements which could be made to improve on existing methods such as considering the impact of including surfactants when testing some chemistries, the usefulness of the PBO synergism assay, and how to interpret and use resistance monitoring data given the high level of variability in bioassays.

A critical priority focus area already identified by the Landscape lies in the validation of well-established methods that have served as the gold standard for many years. For instance, the correlation between the bioavailability of insecticides within products and the mosquito bioassay methods used to assess efficacy demands exploration. There is an assumed link between biological efficacy and product specifications ( Karl *et al.*, 2021), however, this is a critical aspect that needs to be considered when designing effective intervention assessment methods. ITNs vary in material and mechanism of delivery. There is no simple physiochemical measurement available that corresponds to the bioefficacy of treated nets. Vector control product specifications refer to characteristics such as design features ( *i.e.*, fabric choice and weave pattern) and insecticide formulations (insecticide concentration and delivery method) that define a control tool. Product specifications need to ensure that the surface-active ingredient (AI) remains effective for expected duration of a tool’s use, but we do not know if surface concentration always relates directly to insecticide bioavailability. The presentation of an insecticide on a product surface over time is an important factor for consideration, and we currently do not have the methods to investigate this accurately.

Another area of priority to consider is the varying modes of action (MoA) of novel chemistries showing a diverse array of physiological effects on mosquitoes, and the need to have tailored endpoints that conventional assays may not have originally encompassed. We will need to either adapt established methods to account for unique characteristics of novel insecticides or develop new methods from scratch. An ideal bioassay for routine testing of a vector control product would be designed to be globally accessible and scalable to high throughput, with inexpensive equipment, either easily available as with the CDC bottle, provided from a central source as with the WHO tube, or utilising readily available materials. A bioassay would ideally be simple to operate, minimising the specialised training required, and with easily scored endpoints. Standardisation to ensure reproducible data which is between sites and over time, is balanced against robustness against variable testing conditions. A recent example of a novel method is the Ifakara Ambient Chamber Test (I-ACT), which has been developed at Ifakara Health Institute for testing new ITNs ( Kibondo *et al.*, 2022). The I-ACT can measure mortality and blood feeding endpoints and so has uses for both durability and non-inferiority testing ( Massue *et al.*, 2019). Existing protocols are often based on historical data for commonly used insecticides, and there is a lack of baseline data for new products. With this in mind, there is limited information to compare method and product efficacy against. To overcome pyrethroid resistance, new insecticides exploit different target sites and pathways within mosquito populations, which may eventually lead to the emergence of varied resistance mechanisms. Existing methods may not be sensitive to these new modes of resistance, potentially leading to confounding results and underestimation or failure to detect resistance. Additionally, new chemistries may share similarities in their modes of action with existing compounds, leading to potential cross-resistance which we need to have methods in place to detect. I2I see this a critical activity as new products with novel MoA have already gained WHO recommendation for use in areas of pyrethroid resistance, despite there being a lack of suitable and validated laboratory bioassays available for use. New tools may also have different routes of insecticide exposure, oral ingestion, for example, for which new bioassay types may be needed to test durability of bioefficacy as well as susceptibility. We need to work towards incorporating method validation stages into routine product development and use, as opposed to the delayed, reactive approach that has been followed to date ( Lees *et al.*, 2023).

## Call to action

The emergence of insecticide resistance has necessitated the development of novel chemistries and products, rendering previously used methodologies inadequate for the diverse range of tools now available. The development and incorporation of validated methods and consensus standard operating procedures (SOPs) ( Lissenden *et al.*, 2021) will empower the vector control community to confidently use optimised methods that have been tailored for specific insecticides and novel products. It is imperative that the research community work together to address these challenges and prioritise the development, validation, and consistent implementation of improved methods. It is through collective efforts and innovative thinking that we can ensure the reliability of data which forms the evidence base for decisions in vector control during tool development, evaluation, implementation, and post-market monitoring.

## Data Availability

No data are associated with this article.
