# Supplementary material for: Better methods, better data: landscaping the priorities for improving methodologies in vector control
Source: Gates Open Res. 2025 Jul 28;8:27. Originally published 2024 Mar 21. [Version 2] doi: 10.12688/gatesopenres.15399.2 (PMC11259587; doi:10.12688/gatesopenres.15399.2)
Supplement: Peer review history

# Reviewer report: Reviewer response for version 1

**DOI:** 10.21956/gatesopenres.16749.r36726

**Published:** 2024-08-21

**Authors:** Magellan Tchouakui

**Affiliations:** 1 Centre for Research in Infectious Diseases, Yaoundé, Cameroon

Novel insecticides are gradually being recommended by WHO to mitigate pyrethroid resistance.  This highlights the need to review the available testing methodologies and design/improve novel methods for testing the susceptibility of the vectors to these new chemistries. This review reveals a shows insufficiency of validation for many commonly used vector control methods, highlighting the need for improved protocols to enhance the reliability and robustness of the data that is generated to make decisions in product development, evaluation, and implementation. The review is very interesting as it could serve as a decision-making tool for researchers and product manufacturers in selecting appropriate methods and I recommend its acceptance

Does the article adequately reference differing views and opinions?

Yes

Are all factual statements correct, and are statements and arguments made adequately supported by citations?

Yes

Is the Open Letter written in accessible language?

Yes

Where applicable, are recommendations and next steps explained clearly for others to follow?

Yes

Is the rationale for the Open Letter provided in sufficient detail?

Yes

Reviewer Expertise:

Infectious disease transmission, Malaria, Insecticide resistance management, Fitness costs

I confirm that I have read this submission and believe that I have an appropriate level of expertise to confirm that it is of an acceptable scientific standard.

# Response

**Published:** 2025-02-27

**Authors:** Rosemary Lees

**Affiliations:** Liverpool School of Tropical Medicine, UK

We are very grateful for this positive review, and hope that the Methods Landscape will indeed act as a useful decision-making tool as suggested.

# Reviewer report: Reviewer response for version 1

**DOI:** 10.21956/gatesopenres.16749.r36732

**Published:** 2024-07-19

**Authors:** Adedayo O. Oduola

**Affiliations:** 1 Vector Biology and Control Research Group, Department of Zoology, University of Ilorin, Ilorin, Nigeria

The report is timely, relevant  and identifies important gaps in available validation tools for evaluating the performance of insecticide-based vector control interventions. With the emergence of new insecticides with different modes of action, there is a need to develop update existing technology for assessment to be sensitive to the different peculiarities associated with the toxicant and the behavior of the target insects. These are well-known observations that have often led to variations in outcomes.

Does the article adequately reference differing views and opinions?

Yes

Are all factual statements correct, and are statements and arguments made adequately supported by citations?

Yes

Is the Open Letter written in accessible language?

Yes

Where applicable, are recommendations and next steps explained clearly for others to follow?

Yes

Is the rationale for the Open Letter provided in sufficient detail?

Yes

Reviewer Expertise:

Public Health Entomology, Insecticide Resistance Monitoring. Impact Assessment of New Insecticide Tools.

I confirm that I have read this submission and believe that I have an appropriate level of expertise to confirm that it is of an acceptable scientific standard.

# Response

**Published:** 2025-02-27

**Authors:** Rosemary Lees

**Affiliations:** Liverpool School of Tropical Medicine, UK

We thank the reviewer for such a positive response and are pleased that they found this review so relevant.

# Reviewer report: Reviewer response for version 1

**DOI:** 10.21956/gatesopenres.16749.r36733

**Published:** 2024-07-19

**Authors:** Eric Ochomo

**Affiliations:** 1 Kenya Medical Research Institute, Kisumu, Kenya

This is an excellent letter calling for more standardization of the methods available for the evaluation of vector control tools especially as new active ingredients become available. 

 The article does an excellent summary of the history of current basis and does provide a justification to identify new approaches for evaluation of novel actives and standardize those. 

 The only point I felt was not sufficiently emphasized was the need to ensure that such approaches are scalable for the field entomologists who would need to use their to inform their insecticide resistance management.

Does the article adequately reference differing views and opinions?

Yes

Are all factual statements correct, and are statements and arguments made adequately supported by citations?

Yes

Is the Open Letter written in accessible language?

Yes

Where applicable, are recommendations and next steps explained clearly for others to follow?

Yes

Is the rationale for the Open Letter provided in sufficient detail?

Yes

Reviewer Expertise:

Medical entomology, infectious diseases, malaria, vector control

I confirm that I have read this submission and believe that I have an appropriate level of expertise to confirm that it is of an acceptable scientific standard.

# Response

**Published:** 2025-02-27

**Authors:** Rosemary Lees

**Affiliations:** Liverpool School of Tropical Medicine, UK

We thank the reviewer for such a positive response.

 The point about the need for methods to be accessible to their users, who are often field entomologists with finite resources. We have added a paragraph to suggest the key requirements of methods, which includes scalability and affordability.

# Reviewer report: Reviewer response for version 1

**DOI:** 10.21956/gatesopenres.16749.r36730

**Published:** 2024-05-25

**Authors:** Shüné V Oliver

**Affiliations:** 1 National Institute for Communicable Diseases, Johannesburg, South Africa

This letter covers a range of important factors that have long been known but not fully acknowledged by the vector control community. It covers a methodology to identify the sources of variability (and there are many) in the current methodologies used to assess insecticide resistance. The authors cover a range of problems, covering both issues with testing ITNs and IRS agents. The I2I programme therefore represents a critical exercise in the field, and it is critical that this information is disseminated. 

 The information is presented clearly with good examples. There is not much that I can add in terms of language and contextualisation. The only suggestion I can add is that it would be beneficial to add a section on the role of assessing insecticide resistance intensity. Although the methods for assessing intensity is covered already, this is an important factor that is not examined nearly enough. This should be mentioned. It is the only thing that I think that would improve this document.

Does the article adequately reference differing views and opinions?

Yes

Are all factual statements correct, and are statements and arguments made adequately supported by citations?

Yes

Is the Open Letter written in accessible language?

Yes

Where applicable, are recommendations and next steps explained clearly for others to follow?

Yes

Is the rationale for the Open Letter provided in sufficient detail?

Yes

Reviewer Expertise:

Entomology- vector biology, insecticide resistance, environmental effects on insecticide resistance, mosquito microbiomes.

I confirm that I have read this submission and believe that I have an appropriate level of expertise to confirm that it is of an acceptable scientific standard.

# Response

**Published:** 2025-02-27

**Authors:** Rosemary Lees

**Affiliations:** Liverpool School of Tropical Medicine, UK

We thank the reviewer for such positive comments and are glad that they agree that the Methods Landscaping is a useful exercise. We are currently looking into the methods to disseminate the Landscaping and associated resources more widely, beyond making them available on the I2I website: https://innovationtoimpact.org/methodslandscaping/.

 Thank you for the suggestion about adding further discussion on the methods used for insecticide resistance monitoring, which we agree is an important topic. We have added a new section to address this in version 2, and are also planning a future paper focusing on this priority area.
